# A Small Study of Bacterial Contamination of Anaerobic Digestion Materials and Survival in Different Feed Stocks

**DOI:** 10.3390/bioengineering7030116

**Published:** 2020-09-22

**Authors:** Lauren Russell, Paul Whyte, Annetta Zintl, Stephen Gordon, Bryan Markey, Theo de Waal, Enda Cummins, Stephen Nolan, Vincent O’Flaherty, Florence Abram, Karl Richards, Owen Fenton, Declan Bolton

**Affiliations:** 1 Teagasc Food Research Centre, Ashtown, Dublin 15, Ireland; lauren.russell@teagasc.ie; 2 School of Veterinary Medicine, University College Dublin, Belfield, Dublin 4, Ireland; paul.whyte@ucd.ie (P.W.); annetta.zintl@ucd.ie (A.Z.); stephen.gordon@ucd.ie (S.G.); bryan.markey@ucd.ie (B.M.); theo.dewall@ucd.ie (T.d.W.); 3 School of Biosystems and Food Engineering, University College Dublin, Belfield, Dublin 4, Ireland; enda.cummins@ucd.ie; 4 School of Natural Sciences, National University of Ireland, Galway, Ireland; stiofnolan@gmail.com (S.N.); vincent.oflaherty@nuig.ie (V.O.); florence.abram@nuigalway.ie (F.A.); 5 Teagasc Environmental Research Centres, Johnstown Castle, Wexford, Ireland; karl.richards@teagasc.ie (K.R.); owen.fenton@teagasc.ie (O.F.)

**Keywords:** *Salmonella* spp., *Escherichia coli* O157, *Listeria monocytogenes*, *Enterococcus faecalis*, *Clostridium* spp., anaerobic digestion, digestate, pathogens, sustainable farming

## Abstract

If pathogens are present in feedstock materials and survive in anaerobic digestion (AD) formulations at 37 °C, they may also survive the AD process to be disseminated in digestate spread on farmland as a fertilizer. The aim of this study was to investigate the prevalence of *Salmonella* spp., *Escherichia coli* O157, *Listeria monocytogenes*, *Enterococcus faecalis* and *Clostridium* spp. in AD feed and output materials and survival/growth in four formulations based on food waste, bovine slurry and/or grease-trap waste using International Organization for Standardization (ISO) or equivalent methods. The latter was undertaken in 100 mL Ramboldi tubes, incubated at 37 °C for 10 d with surviving cells enumerated periodically and the T_90_ values (time to achieve a 1 log reduction) calculated. The prevalence rates for *Salmonella* spp., *Escherichia coli* O157, *Listeria monocytogenes*, *Enterococcus faecalis* and *Clostridium* spp. were 3, 0, 5, 11 and 10/13 in food waste, 0, 0, 2, 3 and 2/3 in bovine slurry, 1, 0, 8, 7 and 8/8 in the mixing tank, 5, 1, 17, 18 and 17 /19 in raw digestate and 0, 0, 0, 2 and 2/2 in dried digestate, respectively. Depending on the formulation, T_90_ values ranged from 1.5 to 2.8 d, 1.6 to 2.8 d, 3.1 to 23.5 d, 2.2 to 6.6 d and 2.4 to 9.1 d for *Salmonella* Newport, *Escherichia coli* O157, *Listeria monocytogenes*, *Enterococcus faecalis* and *Clostridium sporogenes*, respectively. It was concluded that AD feed materials may be contaminated with a range of bacterial pathogens and *L. monocytogenes* may survive for extended periods in the test formulations incubated at 37 °C.

## 1. Introduction

**Figure 1:**
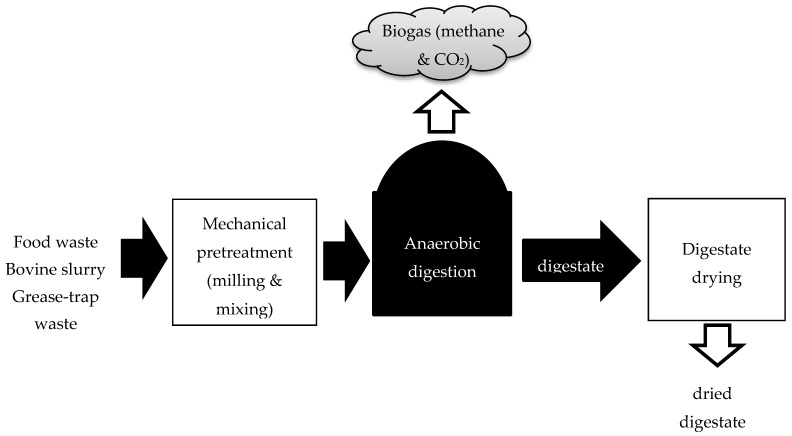
The basic steps in the anaerobic digestion process.

Anaerobic digestion (AD) is a cheap and efficient method for processing the large amounts of organic waste produced by farming (manures and slurries), food processing and sewage treatments (sludge) while contributing to international renewable energy targets. Co-digestion of combined wastes produces biogas (methane and carbon dioxide) and digestate, a nutrient rich fertilizer [1] while recycling nutrients from biowaste back into food production (an important activity in sustainable farming) [2]. In its most basic form, AD involves mechanical pretreatment of the feed waste materials to reduce particle size and mix the formulations, followed by anaerobic digestion, which produces biogas and digestate, the latter of which is usually subject to a treatment (pasteurization or drying) before use as a soil fertilizer (Figure 1). 

There are four stages in anaerobic digestion; hydrolysis, acidogenesis, acetogenesis and methanogenesis [3]. During hydrolysis the lipids, carbohydrates and protein present in the feed materials are broken down into fatty acids, sugars and amino acids, respectively. This is followed by acidogenesis, during which fermentative bacteria produce volatile fatty acids (VFAs), including propionic acid, butyric acid, acetic acid as well as ethanol, ammonia, carbon dioxide and hydrogen sulphide (H_2_S). In the third stage (acetogenesis), the products of acidogenesis are converted into acetic acid, carbon dioxide and hydrogen while during methanogenesis (fourth stage), the products of the preceding stages are converted into methane, carbon dioxide and water [4]. The byproduct, digestate, is a nutrient rich fertilizer.

However, feedstocks may be contaminated with a range of bacterial, viral and parasitic pathogens of veterinary and public health concern [5], which may survive the process, depending on a combination of factors including initial load, feedstock, microbial competition, pH, temperature and ammonia production [6], to be disseminated on farms in contaminated digestate [2,7,8]. Thus, EC Regulations 1069/2009 and 142/2011 require that AD raw materials or digestate must be heat treated at 70 °C or 90 °C for a minimum of 60 min or equivalent. Regardless, it is generally agreed that such treatments are only sufficient to kill vegetative bacteria like *Salmonella*, *Listeria* and *Escherichia coli*, while spore-forming organisms such as *Clostridium* spp. will survive. The application of digestate as a fertiliser is therefore banned in some countries [9].

Farm based AD plants in Ireland currently operate at mesophilic temperatures and typically co-digest animal slurry with food waste [10]. Data on bacterial contamination and survival during the different stages of the AD process is limited. Although the process parameters such as temperature are set to optimise biogas production, other factors such as the composition of feedstock and retention time could be manipulated, if necessary, to promote the destruction of target pathogenic bacteria without negatively impacting on the efficiency of the process [11]. The aims of this study were to test a range of AD input and output materials for the presence of *Salmonella* spp., *E. coli* O157, *L. monocytogenes*, *Enterococcus faecalis* and *Clostridium* spp. and to investigate the survival of representative strains of these bacteria in four AD feedstock materials/formulations, stored at 37 °C in a laboratory-scale batch system previously used in similar studies [12].

## 2. Materials and Methods

### 2.1. Pathogen Evaluation/Survey

#### 2.1.1. AD Samples

Food waste (a mixture of dairy and vegetable wastes; *n* = 13), bovine slurry (*n* = 3), mixing tank (*n* = 8), raw digestate (*n* = 19) and dried digestate (*n* = 2) samples were collected from 3 separate commercial AD facilities located in the east of Ireland. These materials were not preselected but were the feedstock materials being used on the day of each visit. Each plant was visited on one occasion and the samples aseptically removed using a sterile scoop (Sterileware, Fisher Scientific Ireland, Dublin, Ireland) and sterile containers (VWR, Dublin, Ireland). All samples were transported to the laboratory in a cool box at 2–4 °C within 3 h.

#### 2.1.2. Microbiological Analysis

Exactly 25 g of each sample was diluted and/or enriched in 225 mL of diluent or broth before plating on selective agar and incubated at 37 °C for 24 h, unless otherwise indicated (Table 1). Presumptive colonies were confirmed using culture based and PCR methods (also Table 1). All media (except BBL Enterococcosel broth, which was supplied by Becton Dickinson (Limerick, Ireland)) were Oxoid products and purchased from Fannin Ltd., (Dublin, Ireland), as were the AnaeroGen sachets. Immunomagnetic separation (IMS) beads by Dynal^®^ BeadRetriever were supplied by Thermo Fisher Scientific (Dublin, Ireland) while the Sifin anti-coli O157 sera test and defibrinated horse blood were provided by Cruinn Diagnostics Ltd., (Dublin, Ireland).

### 2.2. Survival Studies

#### 2.2.1. Inoculum Preparation

*Salmonella* Newport, *E. coli* O157 (NCTC 12900), *L. monocytogenes* and *E. faecalis* (NCTC 12697) strains were obtained from the Teagasc culture collection. The *S.* Newport and *L. monocytogenes* strains had a streptomycin resistance (1000 µg/mL) marker to facilitate recovery. To prepare the inoculum, a culture bead from frozen storage was streaked on TSA and incubated at 37 °C for 24 h. A single colony was then selected and placed into 10 mL of tryptone soya broth (TSB; Oxoid, Fannin Ltd., Ireland) and incubated overnight at 37 °C. The culture obtained was centrifuged and washed 3 times with phosphate buffered saline (PBS; Oxoid, Fannin Ltd., Ireland), before resuspension in PBS and serially diluted to obtain a cell concentration of approximately 10^5^ cfu/mL.

Freeze-dried *C. sporogenes* DSM 767 obtained from the Deutsche Sammlung von Mikroorganismen und Zellkulturen (DSMZ, Braunschweig, Germany) were rehydrated as per the instructions provided. Twenty tubes of cooked meat medium (CMM; Oxoid, Fannin Ltd., Ireland) broth (20 mL) were inoculated with 100 µL rehydrated *C. sporogenes*, and incubated in an anaerobic cabinet for 12–18 h at 37 °C. Clostridium sporulation agar was prepared as described by [18] and placed in a Whitley A35 anaerobic chamber (Don Whitley Scientific, West Yorkshire, UK) overnight using the ANO_2_ gas mixture (10% H_2_, 10% CO_2_ and 80% N_2_; Air Products Ireland, Dublin, Ireland) to exclude all oxygen. Aliquots (300 µL) of the overnight CMM broth were then spread onto 300 plates of CSA (inside the anaerobic chamber) before transfer to anaerobic boxes (GenBOX jars; BioMérieux UK Ltd., Basingstoke, UK; AnaeroGen sachets; Oxoid, Fannin Ltd., Ireland) and incubated at 37 °C for 12 d. The CSA plates were then inspected to ensure sufficient spore growth for harvesting. Spore harvesting took place in a laminar flow hood. Approximately 4–5 mL ice-cold sterile distilled water was placed onto the surface of the CSA plates, agitating the surface of the agar with a sterile spreader to release the spores. The suspension was then transferred to the next agar plate and the scraping process repeated. This method was repeated until spores had been harvested from all of the 300 CSA plates. The suspensions were pooled in 50 mL tubes, centrifuged at 7000 RPM at 4 °C for 10 min and washed with iced water, reducing the amount of liquid over the course of repeated cycles until a spore suspension of approximately 10^7^ spores/mL (estimated by phase contrast microscope examination), which was then confirmed by plating out on Columbia blood agar (CBA; Oxoid, Fannin Ltd., Ireland) with 5% defibrinated horse blood (Cruinn diagnostics, Ireland). The spore preparations (1 mL aliquots) were stored at −80 °C. Prior to inoculation, spore preparations were thawed at room temperature, prior to heat treatment at 80 °C for 10 min to ensure the exclusion of vegetative cells.

#### 2.2.2. AD Commercial Formulation Preparation

Four feedstock mixtures; [1] 100% food waste (primarily vegetable matter with small amounts of cooked meats and bakery product waste); [2] slurry (bovine) and food waste (1:3); [3] slurry and food waste (3:1) and [4] slurry and grease-trap waste (from restaurants) (2:1) were formulated on a volumetric basis as per the advice of our commercial AD stakeholders. Food waste was supplied by local restaurants, slurry by beef farms in counties Galway, Louth and Meath and grease-trap waste from the Bioenergy and Organic Fertilizer Services (BEOFS) AD plant in Camphill, County Kilkenny, Ireland. Before use all samples were tested to ensure the target bacteria were absent.

#### 2.2.3. The Laboratory Model System

Exactly 70 model reactors were prepared for each of the four mixtures. Each contained 10 mL of fresh seed material (obtained from a commercial AD bioreactor) mixed with 20 mL of the feedstock material in a sterile 100 mL tube (Ramboldi tubes, VWR, Ireland). For each mixture, 14 tubes were randomly assigned to each of the bacteria being studied. The bacterial cells/spores, prepared as described above, were then added to 1 mL MRD to give a final concentration of approximately 10^4^ cells or approximately 10^7^ spores/mL. The tubes were then incubated anaerobically (GenBOX jars; bioMérieux UK Ltd., Basingstoke, UK; AnaeroGen sachets; Oxoid, Fannin Ltd., Ireland) at 37 °C. Duplicate tubes were removed periodically (0 (immediately after inoculation), 1, 2, 3, 4, 5 and 10 d), from the vortexed tubes, the pH recorded (Eutech pH 150 probe (Thermo Scientific, Waltham, MA, USA), which was calibrated using pH 4, 7 and 10 standards prior to use) and the surviving cells/spores enumerated. 

#### 2.2.4. Enumeration of Surviving Cells

The extracted samples (1 mL) were diluted in 9 mL MRD and serial dilutions prepared. Surviving cells/spores were enumerated as described in Table 2. All media and the AnaeroGen sachets were Oxoid products and purchased from Fannin Ltd., (Dublin, Ireland). Streptomycin sulphate was obtained from Sigma Aldrich Ireland Ltd., (Wicklow, Ireland). Agar plates were incubated at 37 °C for 24 h, unless otherwise indicated.

### 2.3. Data Analysis

The survival study, as described above, was performed in duplicate and repeated on three separate occasions. Bacterial counts were converted into log_10_ cfu/mL and the T_90_-values (the time required to achieve a 90% (1 log) reduction in the population) were determined by linear regression using GraphPad Prism 7 software (San Diego, CA, USA), considering each replicate Y-value as an individual point. Differences between slopes were examined using ANOVA and Tukey’s multiple comparison tests (GraphPad Prism 7.02). Statistical significance was set at the 5% level (*p* < 0.05).

## 3. Results

The results of the survey of commercial AD inputs and outputs are shown in Table 3. *Salmonella* spp. were detected in the food waste (3 positive out of 13 samples tested (3/13)), mixing tank (1/8) and raw digestate (5/19) samples. *E. coli* O157 was only detected in one sample (raw digestate). In contrast *L. monocytogenes*, *E. faecalis* and *Clostridium* spp. were common in food waste (5, 11 and 10/13), slurry (2, 3 and 2/3), mixing tank (8, 7 and 8/8) and raw digestate (17, 18 and 17/19) samples. The latter two bacteria were also detected in the two dried digestate samples tested.

In the model 100 mL tubes, the pH of the food waste (100%) and slurry and food waste (1:3) formulations decreased from pH 7.1 to 5.8. and from pH 7.2 to 6.0, respectively (data not shown). In contrast the pH values in the slurry and food waste (3:1) increased from pH 7.5 to 8.0 while the pH was stable at pH 8.0 in the slurry and grease-trap waste (2:1) over the 10 d of the study. 

**Figure 2:**
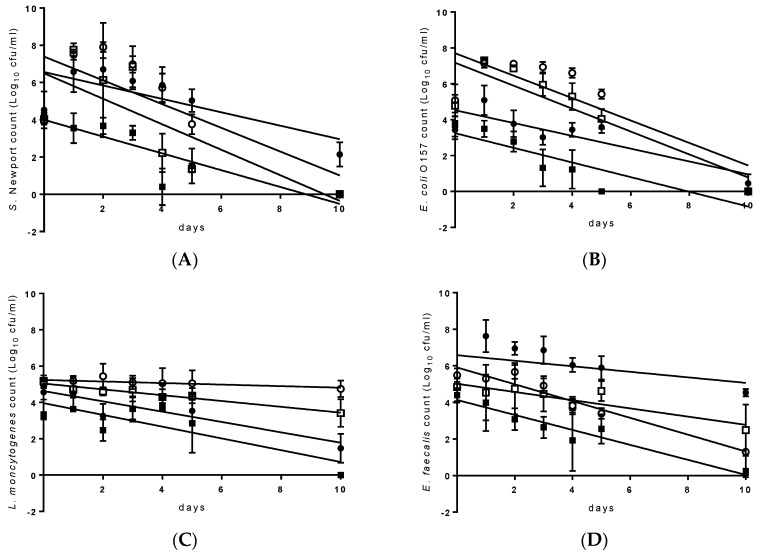

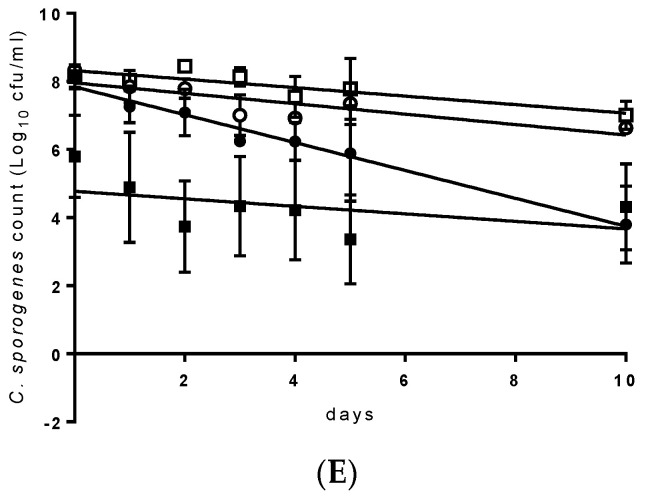
Linear regression graphs for *Salmonella* Newport (**A**), *Escherichia coli* O157 (**B**), *Listeria monocytogenes* (**C**), *Enterococcus faecalis* (**D**) and *Clostridium sporogenes* (**E**), in food waste (□), slurry and food waste (1:3) (○); slurry and food waste (3:1) (●) and slurry and grease-trap waste (2:1) (■). Each point is the mean of 6 data points (*n* = 6) and the error bar is the standard deviation.

The results of the regression analysis are provided in Figure 2 and Table 4. An initial period of growth (1–3 d) was observed in food waste (100%; *S*. Newport, *E. coli* O157 and *C. sporogenes*), slurry and food waste (1:3; *S*. Newport, *E. coli* O157 and *E. faecalis*), slurry and food waste (3:1; *S*. Newport and *E. faecalis*) and in slurry and grease-trap waste (2:1; *E. coli* O157). The time required toachieve a 1 log reduction in the *S*. Newport and *E. coli* O157 populations ranged from 1.5–2.8 d, with significantly (*p* < 0.05) higher T_90_-values observed for slurry when combined with food (3:1) and grease-trap waste (2:1). In contrast, the T_90_-values for *L. monocytogenes* were significantly lower in these two formulations (3.5 and 3.1 d, respectively) as compared to those obtained for the same bacteria in food waste (6.2 d) and slurry and food waste (1:3). The latter provided an environment where any reduction was minimal (slope = 0.04), resulting in an estimated 23.5 d required to achieve a 90% population reduction. T_90_-values for *E. faecalis* ranged from 2.2 to 6.6 d with the latter obtained in slurry and food waste (3:1). *C. sporogenes* T_90_-values ranged from 2.4 to 9.1 d, with significantly different values obtained in each of the formulations in the order of; slurry and grease-trap waste (2:1) > food waste > slurry and food waste (1:3) > slurry and food waste (3:1).

## 4. Discussion

The commercial AD feedstock samples (food waste, bovine slurry and mixing tank materials) were contaminated with pathogens of public health significance including *Salmonella* spp., *L. monocytogenes*, *E. faecalis* and *Clostridium* spp. but not *E. coli* O157. Although there is little or no data for food waste or mixing tank materials, bovine faeces has been extensively tested and previous Irish studies have reported *Salmonella* spp. and *L. monocytogenes* contamination rates of 2–3% [20,21] and 5–12% [21,22], respectively, while 0.7–2.4% of samples are contaminated with *E. coli* O157 [23,24].

*Salmonella* and *Clostridium* spp. have also been detected in other AD feed materials [5,7,25]. To the best of our knowledge this is the first study reporting the presence of *L. monocytogenes* and *E. faecalis*, but this was not unexpected as these bacteria are widespread in the natural environment [26]. Of greater concern was the presence of all the target bacteria, including *Salmonella* spp. and *E. coli* O157, in raw digestate. *Salmonella* has been previously detected in digestate, suggesting these bacteria survive the AD process [5,7], although the possibility of post-reactor contamination cannot be ruled out. In contrast, only *E. faecalis* and *Clostridium* spp. were detected in the dried digestate, suggesting the drying process is sufficient to kill most but not all the bacteria of concern. This is an important finding, as several countries (including Ireland), have a standard requirement for the absence of *Salmonella* in 25 g before this material can be used as a fertiliser [7].

This study also investigated the survival of *Salmonella* spp., *E. coli* O157, *L. monocytogenes*, *E. faecalis* and *Clostridium* spp. in four AD feedstock formulations at 37 °C in a small scale laboratory system. Although previously shown to be a useful study tool [12], laboratory-scale batch systems may not be representative of full-scale continuous commercial bioreactors due to differences in inoculation methods, rheology and hydrodynamic factors [27]. Moreover, as our feedstock mixtures were formulated on a volumetric basis, it is possible that the organic load could have been different between the various formulations. This would affect the production of VFAs, ethanol, ammonia, hydrogen disulphide, etc., by the bacteria present, thereby influencing pathogen survival. Thus, while the survival data obtained provides a good indication of the relative resistance of each bacteria in the materials and under the conditions tested, further research would be required to obtain a more accurate representation of how these organisms behave in large scale commercial systems.

The T_90_-values for *S*. Newport ranged from 1.5 to 2.8 d, regardless of the feed stock formulation. Interestingly, these values are similar to those previously reported for the decline of *Salmonella* spp. in the initial stages of the AD process, which typically range from 0.2 d in sewage sludge [28] to 7 d in a mixture of plant waste, cattle manure and cattle slurry [29,30,31]. The *E. coli* O157 T_90_ values (1.6–2.8 d) were similar to those of *S.* Newport and within the range of 0.5–6.5 d reported in previous AD pathogen survival studies [31,32,33,34,35]. Considering these bacteria survive for extended periods (at least 3 months) in bovine slurry [36,37] our data supports the hypothesis that AD is an effective process for *Salmonella* and *E. coli* O157 removal from animal waste.

In three of the four formulations the population of *L. monocytogenes* decreased by 1 log_10_ cfu/mL after approximately 3–6 d but in slurry and food waste (1:3) the population was almost stable resulting in a regression slope close to zero (−0.04). While previous studies have reported typical T_90_-values of 1.5–2.2 d, in AD formulations [38,39,40], *L. monocytogenes* may also achieve a steady state during AD where the population is maintained for extended periods and the T_90_ values are as high as 12.3 d in batch slurry and 35.7 d in semi-continuous digestion. This is not unexpected as *L. monocytogenes* have a host of molecular mechanisms that facilitate survival in a range of different environments [41]. The T_90_-value for *E. faecalis* ranged from 2.2 to 6.6 d, with significantly higher endurance in food waste and in slurry and food waste (3:1). These values compare to the 0.1–7 d previously reported for *Enterococcus* spp. in different feed-stocks (dairy waste, cattle slurry, swine manure and sewage sludge) [31,32,33,35,40,42,43] and is of particular significance as enterococci are considered to be good indicators of the fate of bacterial vegetative cells during AD [43]. *C. sporogenes* survival rates were lower than expected, with T_90_ values of 2.4–9.1 d. While comparable data for *C. sporogenes* is not available, Froschle et al. [25] found it required approximately 35 d to achieve a 1 log reduction in the population of *Clostridium botulinum* in laboratory scale digesters at 38 °C, while Chauret et al. [40] observed no change in the concentration of *C. perfringens* in the mesophilic digestion of sewage sludge after 20 d. Our observations are inconsistent with these findings and may be the result of the experimental design, for example elevated carbohydrate concentrations stimulating early VFA production, but further investigation is required.

When the different formulations were compared the results were mixed and there was no one mixture that consistently provided higher or lower T_90_ values for all of the bacteria tested. Food waste, alone and when combined with slurry, supported an initial growth phase (1 d) for *S.* Newport, *E. coli* O157 and/or *E. faecalis*, which are metabolically similar under anaerobic conditions, but also provided the lowest T_90_-values for these bacteria. Interestingly, increasing the proportion of slurry in these mixtures resulted in significantly higher T_90_-values for these bacteria but the opposite was observed with *L. monocytogenes* and *C. sporogenes*. Thus, while the bacteria tested decreased, the reduction rate was dependent on factors other than the formulation, as previously reported [44].

## 5. Conclusions

It was concluded that AD feed materials might be contaminated with a range of bacterial pathogens. However given the large volumes used in commercial bioreactors these would be diluted out and present at very low concentrations. In the laboratory-scale batch system used in our experiments, the survival rates of *S.* Newport, *E. coli* O157 and *E. faecalis* were similar to those previously reported while *C. sporogenes* declined more rapidly than expected. This requires further investigation as does the ability of *L. monocytogenes* to survive for extended periods during AD, perhaps necessitating mandatory pasteurisation of digestate.

## Figures and Tables

**Table 1:** The isolation and confirmation methods used to test the samples for the target bacteria.

Detection		Confirmation	
Treatment	Selective Agar	Culture Based	Molecular
*Salmonella* spp.			
buffered peptone water	modified semi-solid Rappaport Vassiliadis medium with novobiocin supplement (20 mg/L), incubated at 42 °C for 24 h	Xylose lysine deoxycholate (XLD) agar	Pathmanathan et al. [13]
*E. coli* O157			
modified tryptone soya broth (mTSB) containing cefixime (50 µg/L) and vancomycin (6 mg/L)	Immunomagnetic separation with plating on sorbitol MacConkey agar supplemented with cefixime-tellurite (CT-SMAC)	Eosin methyl blue agar and plate count agar (PCA) followed by agglutination testing using the Sifin anti-coli O157 sera test	Paton and Paton [14].
*L. monocytogenes*			
half strength Fraser broth, incubated overnight at 30 °C followed by full strength Fraser broth incubated at 37 °C for 48 h	*Listeria* Selective Oxford agar and Brilliance *Listeria* agar (BLA), incubated at 37 °C for 48 h	PCA	Terzi et al. [15]
*E. faecalis*			
BBL Enterococcosel broth and plated on Slanetz and Bartley agar (SBA) incubated at 37 °C for 24 h, followed by 44 °C for an additional 24 h	Pink colonies were streaked on PCA and stabbed in rows into well-dried bile aesculin agar plates, incubated at 44 °C for 24 h.	PCA	Dutka-Malen et al. [16]
*Clostridium* spp.			
Maximum recovery diluent before plating on reinforced clostridial agar (RCA) incubated anaerobically (AnaeroGen sachets in BioMérieux GENbox jars (Hampshire, UK) at 37 °C for 48 h	Columbia blood agar supplemented with 5% defibrinated horse blood		Song et al. [17]

**Table 2:** Methods for enumerating surviving cells or spores.

	Enumeration	PCR Confirmation
*S.* Newport	XLD, supplemented with streptomycin sulphate (1000 µL/g)	Pathmanathan et al. [13]
*E. coli* O157	CT-SMAC	Paton and Paton [14].
*L. monocytogenes*	BLA, supplemented with streptomycin sulphate (1000 µL/g) incubated at 37 °C for 48 h	Terzi et al. [15].
*E. faecalis*	SBA incubated at 37 °C for 24 h, followed by 44 °C for a further 24 h	Dutka-Malen et al. [16].
*C. sporogenes*	RCA, incubated anaerobically (AnaeroGen sachets in BioMérieux GENbox jars (Hampshire, UK) at 37 °C for 48 h	Song et al. [17] and Morandi et al. [19].

**Table 3:** Detection of the target pathogens in the different types of samples.

Pathogen	*Salmonella* spp.	*E. coli* O157	*L. monocytogenes*	*E. faecalis*	*Clostridium* spp.
Type of samples					
Pre anaerobic digestion					
food waste (13) ^1^	Positive (3) ^2^	negative	positive (5)	positive (11)	positive (10)
bovine slurry (3)	negative	negative	positive (2)	positive (3)	positive (2)
mixing tank (8)	positive (1)	negative	positive (8)	positive (7)	positive (8)
Post anaerobic digestion					
raw digestate (19)	positive (5)	positive (1)	positive (17)	positive (18)	positive (17)
dried digestate (2)	negative	negative	negative	positive (2)	positive (2)

^1^ total number of samples tested; ^2^ number of positive samples.

**Table 4:** Observed growth and decay rate (T_90_-values; the time for the bacterial concentration to decrease by 1 log unit) for the 5 pathogens (*Salmonella* spp., *Escherichia coli* O157, *Listeria monocytogenes*, *Enterococcus faecalis* and *C*. *sporogenes*) in the 4 different AD feedstock recipes.

Pathogen	Recipe	Growth			Decay Rate				
		Yes/No	Period	Maximum Concentration (log_10_ cfu/mL)	Slope	SE	R^2^- Value	T_90_-Value (d)	*n*
*S.* Newport	^1^ FW	yes	1d	7.8	−0.69	0.110	0.49	1.5 ^A^	42
	^2^ SF1	yes	1d	7.3	−0.64	0.089	0.56	1.6 ^A^	42
	^3^ SF2	yes	1d	6.7	−0.36	0.029	0.45	2.8 ^B^	42
	^4^ SGW	no	^5^ NA	^6^ NA	−0.45	0.051	0.66	2.2 ^B^	42
*E. coli* O157	FW	yes	1d	7.3	−0.64	0.062	0.77	1.6 ^A^	42
	SF1	yes	1d	7.2	−0.63	0.073	0.64	1.6 ^A^	42
	SF2	no	ND	NA	−0.36	0.044	0.62	2.8 ^B^	42
	SGW	yes	1d	5.1	−0.41	0.049	0.64	2.5 ^B^	42
*L. monocytogenes*	FW	no	ND	NA	−0.16	0.016	0.49	6.2 ^B^	42
	SF1	no	ND	NA	^7^ −0.04	0.027	0.05	23.5 ^C^	42
	SF2	no	ND	NA	−0.28	0.039	0.77	3.5 ^A^	42
	SGW	no	ND	NA	−0.32	0.050	0.51	3.1 ^A^	42
*E. faecalis*	FW	no	ND	NA	−0.22	0.053	0.31	4.5 ^B^	42
	SF1	yes	1d	7.6	−0.46	0.030	0.85	2.2 ^A^	42
	SF2	yes	1d	7.6	−0.15	0.060	0.14	6.6 ^C^	42
	SGW	no	ND	NA	−0.41	0.049	0.63	2.4 ^A^	42
*C. sporogenes*	FW	yes	3d	7.1	−0.13	0.025	0.38	8.0 ^C^	42
	SF1	no	ND	NA	−0.15	0.024	0.50	6.5 ^B^	42
	SF2	no	ND	NA	−0.41	0.039	0.74	2.4 ^A^	42
	SGW	no	ND	NA	−0.11	0.073	0.54	9.1 ^D^	42

^1^ FW = food waste; ^2^ SF1 = slurry and food waste (1:3); ^3^ SF2 = slurry and food waste (3:1); ^4^ SGW = slurry and grease-trap waste (2:1); ^5^ ND = not detected; ^6^ NA = not applicable; ^7^ slope is very close to zero (0.04) hence the R^2^ value is almost zero. Statistical analysis: for a given bacteria a different capital letter (A, B, C or D) indicates significantly different T_90_-values at the 5% level (*p* < 0.05).
